# Effect of slurry ice during storage on myofibrillar protein of *Pseudosciaena crocea*

**DOI:** 10.1002/fsn3.2355

**Published:** 2021-06-04

**Authors:** Feng Guan, Yirui Chen, Simin Zhao, Zhuo Chen, Chen Yu, Yongjun Yuan

**Affiliations:** ^1^ College of Biological and Environmental Sciences Zhejiang Wanli University Ningbo P.R. China; ^2^ Department of Genetics, Bioinformatics and Computational Biology Virginia Polytechnic Institute and State University Blacksburg VA USA

**Keywords:** crushed ice, fish refrigeration, myofibrillar protein, *Pseudosciaena crocea*, slurry ice

## Abstract

In order to explore the effect of slurry ice on myofibrillar protein of *Pseudosciaena crocea*, the changes in myofibrillar protein and muscle microstructure during storage were studied with crushed ice as a control. During the storage period, the rate of decrease in myofibrillar protein content, Ca^2+^‐ATPase activity, and total sulfhydryl groups in the slurry ice group was lower than in the control group (*p* < .05). There was a significant linear correlation between the hydrophobicity and the storage time (*R*_crushed ice (4℃)_ = 0.9881, *R*_slurry ice (4℃)_ = 0.9878, *R*_slurry ice (−1℃)_ = 0.9674), and trichloroacetic acid (TCA) soluble peptide content was lower than in the control group at the same time. Slurry ice (−1℃) was optimal in maintaining protein content in *P. crocea*; the arrangement of myofibrils in *P. crocea* treated by slurry ice was compact and the gaps were small. Slurry ice can delay the denaturation and degradation of fish myofibrillar protein and maintain its quality.

## Graphical abstract

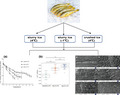

**Highlights**

- Slurry ice can delay the denaturation and degradation of fish myofibrillar protein.
- Slurry ice (−1℃) is the best one to keep protein of *Pseudosciaena* *crocea*.
- There was a linear correlation between the hydrophobicity and the storage time.

## 1 INTRODUCTION

*Pseudosciaena crocea* is an economically important marine fish that is widely cultured in southeast China due to its pleasant taste and high nutritional value (Liu & Mitcheson, 2008). In China, 165,496 tons of this fish species were farmed in 2016, placing it first for mariculture output (Guo et al., 2017).

Fish and fishery products have long been recognized as healthy foods with excellent nutritional value, providing high‐quality protein, minerals, vitamins, essential fatty acids, and trace elements. Fish is widely consumed in many parts of the world by humans due to the high content of good protein characterized by an excellent amino acid composition and easy digestibility (Venugopal et al., 1996). Freezing is a preferred technique to preserve fish and fish products for extended periods. It preserves the flavor and nutritional properties better than storage above the initial freezing temperature. It also has the advantage of minimizing microbial and enzymatic activities (Martino et al., 1998).

Slurry ice, also known as fluid ice, ice mud, and liquid ice, is a mixture of fine ice crystals with a diameter of 0.1–1 mm and a carrier liquid (Kauffeld et al., 2005). It represents a new type of green preservation technology. Slurry ice crystals are small and smooth, fluid, and do not cause mechanical damage to the product. It can be pumped through pipelines, and there are no bubbles between the product and the slurry ice (Bellas et al., 2002). It can be stored and transported in a fully enclosed manner, has the advantages of environmental protection and cleanliness, is widely used in food processing and preservation (Kauffeld et al., 2010; Losada et al., 2007; Narasimha Murthy et al., 2017), ice storage air conditioning (Bellas & Tasso, 2005), medical treatment (Laven et al., 2007), and other fields. Slurry ice has a high surface heat exchange rate, can quickly cool aquatic products (Pineiro et al., 2004), can inhibit the growth and reproduction of spoilage microorganisms in aquatic products, inactivate biochemical reactions in the body, maximize the quality of aquatic products, and extend shelf life. Therefore, there are many advantages for application in the field of aquatic product preservation. Previous studies on the preservation of aquatic products such as squid (Narasimha Murthy et al., 2017), barramundi (Zakhariya et al., 2015), bonito (Zhang et al., 2015), and other aquatic products, have mainly focused on sensory characteristics, microbial indicators, and chemical indicators. However, little research has been done on the changes in myofibrillar protein in fish muscle during storage.

Protein is one of the most important nutritional components of *P*. *crocea*. Structural changes in the protein occur under different storage conditions; changes to the proteins can result in a dull and opaque texture, and the tissue becomes soft and spongy, severely affecting the quality of the fish product (Schilling et al., 2017). Therefore, protein degradation in aquatic products such as fish can affect freshness, and it is pertinent to study the changes in myofibrillar proteins. Previously we investigated the precooling effects of slurry ice and the changes in sensory quality, texture characteristics, microbial, and chemical parameters of *P*. *crocea* (large yellow croaker fish) during storage. In this study, slurry ice with fine particles and good fluidity was used as the preservation medium, and the effects on changes in myofibrillar protein and muscle tissue microstructure were investigated in comparison with traditional crushed ice refrigeration.

## 2 MATERIALS AND METHODS

### 2.1 Materials

*Pseudosciaena crocea* were cultured in the northern shore sea area of Xiangshan Port (N 29.31°, E 121.33°). After capture, they were aerated and transported to the Marine and Fishery Science and Technology Innovation Base (Ningbo City) for treatment. The mass of each fish was determined to be 500 ± 50 g.

Tris‐hydrochloride (Tris‐HCl), ammonium molybdate, anhydrous ethanol, anhydrous sodium sulfite, ammonium persulfate, sodium hydroxide, hydroquinone, and glutaraldehyde were analytically pure and purchased from Sinopharm Chemical Reagent Co., Ltd; sodium dodecyl sulfate, bovine serum albumin, and tyrosine were analytically pure and purchased from Beijing Solaibao Technology Co., Ltd.

### 2.2 Grouping and treatment

Seawater (3.3% salt content) was added directly to the insulation bucket, the condensate water and solenoid valve were opened, and the liquid icemaker started to prepare delicate slurry ice with good fluidity. The system temperature was −2.2℃ and the mass ratio of micro‐ice particles and water in the system was 7:3. The *P*. *crocea* were completely submerged in slurry ice, sealed, and stored in a refrigerator at either 4℃ or −1℃. A control group using broken ice was used to store the *P*. *crocea* in a “layer‐ice‐fish” manner and sealed and stored in a refrigerator at 4℃. Freshly fished *P*. *crocea* without cold treatment were used as a zero‐day storage sample. This was sampled every other day for 6 days after low‐temperature storage, and subsequently every day. The samples were used for the determination of various protein parameters and the observation of muscle microstructure. Each sample contained three replicates, which were used to determine the mean value.

### 2.3 Extraction of myofibrillar proteins

Myofibrillar proteins were prepared using the method of Saeki (1997), with modifications. Briefly, an appropriate amount of fish was weighed and incubated with four volumes of precooling buffer A (20 mmol/L Tris‐HCl buffer containing 0.1 mol/L KCl; pH 7.5), homogenized, and then centrifuged at 10,000 ✕ *g* for 20 min at 4℃. The supernatant was discarded, and this was repeated twice. Subsequently, four volumes of precooling buffer B (20 mmol/L Tris‐HCl buffer containing 0.6 mol/L KCl; pH 7.0) were added to the precipitate, homogenized for 2 min, and then incubated at 4℃ for 1 hr. This was then centrifuged at 10,000 ✕ *g* for 15 min, and the supernatant containing the myofibrillar protein solution was retained. The myofibrillar protein concentration was determined using the biuret method (Itzhaki & Gill, 1964).

### 2.4 Determination of total sulfhydryl content of myofibrillar proteins

Sulfhydryl content was determined according to the method of Ellman (1959) with slight modifications. Briefly, 1.0 ml of myofibrillar protein solution was added to 9.0 ml of 0.2 mol/L Tris‐HCl buffer (containing 8.0 mol/L urea, 2% SDS, 10 mmol/L EDTA; pH 6.8). After thorough mixing, 4.0 ml to 0.4 ml of 0.1% DTNB solution was added, and the solution placed in a 40℃ water bath for 25 min. Absorbance was measured at 412 nm, using a 0.6 mol/L KCI solution as the blank. The total sulfhydryl content was calculated as follows:

(1) $$C_{0}\;=\;\left(A\times D\right)/\zeta \;\times \;\rho ,$$

where, C_0_ is the sulfhydryl molar concentration; A is the absorbance at the wavelength of 412 nm; D is the dilution factor; ζ is the molecular absorption coefficient of 13,600 (mol·cm/L); ρ is the protein concentration (mg/mL).

### 2.5 Determination of myofibrillar protein Ca^2+^‐ATPase activity

Solution C (0.5 mol/L Tris‐maleic acid buffer, pH 7.0; 0.1 mol/L CaCl_2_ aqueous solution, pH 7.0; 20 mol/L ATP solution) was added to the myofibrillar protein solution and incubated in a water bath at 30℃ for 5 min. Trichloroacetic acid (TCA) solution (1.0 ml of 15% w/v) was then added to stop the reaction. For the blank control, 1.0 ml of 15% (w/v) TCA solution was added immediately before the mixture reaction to denature the protein. After the reaction, 1 ml solution was added to 1 ml molybdic acid sulfate solution, 0.5 ml metal reagent, and 2.5 ml distilled water, mixed, and reacted at 25℃ for 45 min. The absorbance was measured at a wavelength of 640 nm. Ca^2+^‐ATPase activity was calculated as follows:

(2) $${\text{Ca}}^{2+}‐\text{ATPase activity}\;=\;\left(A‐B\right)/t\;\times \;\text{amount of enzyme protein},$$

where: A is the amount of phosphoric acid generated by 1 ml reaction solution, µmol; B is the blank value, µmol; t is the reaction time; and the amount of enzyme protein is the amount of enzyme contained in 1 ml reaction solution, mg.

### 2.6 Determination of surface hydrophobicity of myofibrillar proteins

Surface hydrophobicity was determined using the method of Chelh et al., (2006). The protein concentration was adjusted to 5 mg/ml with 0.02 mol/L phosphate buffer, pH 6.0. The protein solution (1 ml) was added to a centrifuge tube and 200 μL of 1 mg/ml bromophenol blue added. The solution was left at room temperature for 10 min, before centrifugation at 6,000 ✕ *g* for 10 min. The supernatant was diluted 10‐fold and the absorbance measured at 595 nm. The results were calculated as follows.

(3) $$\text{Bromophenol blue binding amount}/\mu g\;=\;200\;\times \;\left(A_{0}‐A\right)/A_{0},$$

where: A_0_ is the blank absorbance; A is the sample absorbance.

### 2.7 Determination of TCA soluble peptides

The content of TCA soluble peptides was determined using the Lowry method, with reference to the method of Morrissey et al., (1993). A standard curve was prepared using tyrosine solutions with concentrations of 0, 20, 40, 60, 80, and 100 μg/ml. Tyrosine solution (1 ml) was added to 1 ml of diluted Folin reagent, and 5 ml of 0.8 M Na_2_CO_3_, mixed, and incubated at 40℃ for 20 min. The absorbance was then measured with a microplate reader (Thermo Fisher Scientific) at a wavelength of 660 nm.

Fish meat (3.0 g) was added to 27 ml of 50 g/L TCA, homogenized for 1 min, allowed to stand in an ice bath for 1 hr, and centrifuged at 5,000 × g for 5 min at 4℃. The supernatant (1 ml) was treated as described above in place of the tyrosine solution. The amount of tyrosine released was calculated by referring to the standard curve, and the result was expressed in µmol/g.

### 2.8 Scanning electron microscopy (*SEM*) observation of microstructure

*SEM* was performed according to the method of Zhou et al., (2019) with slight modifications. The cultured *P*. *crocea* was collected under different storage conditions, and the back muscles were cut into 3.0 mm × 3.0 mm × 1.5 mm cuboids from the same position in each sample. The sections were fixed with 2.5% (v/v) glutaraldehyde for 24 hr, the morphological changes of mycelium were observed by *SEM* (Hitachi Limited).

### 2.9 Statistical analysis

The data analysis followed a completely randomized design (Kuehl, 2000) in Python software (version 3.7, Python Institute). To avoid the effect of storage time variable, we firstly utilized Equation (4) to normalize the data.

(4) $$\hat{y}=\frac{y‐y_{0}}{d}$$

(5) $$Y_{\mathrm{ijk}}=\mu +\tau_{i}+\beta_{j}+{\left(\tau \beta \right)}_{\mathrm{ij}}+\epsilon_{\mathrm{ijk}};i=1,2;j=1,2;k=1,2,3$$

where, y is the response, y_0_ represents the response from Day 0, and d represents the storage time.

In this work, we estimated the effect of treatments on five responses; TCA soluble peptides, Ca^2+^‐ATPase, total sulfhydryl, myofibrillar protein, and surface hydrophobicity. The following treatment conditions were tested: 4℃ crushed ice, 4℃ slurry ice, and −1℃ slurry ice, with three replicates per treatment. Equation (4) shows the linear model used for ANOVA analysis. Here, *Y_ijk_* is the normalized response (TCA soluble peptides, Ca^2+^‐ATPase, total sulfhydryl, myofibrillar protein and surface hydrophobicity), *μ* is an overall mean, *τ_i_* is the effect of temperature, *β_j_* is the effect of ice types, and *ϵ_ijk_* is the experimental random error. To conduct the pairwise comparison, we used a *t* test with Bonferroni correction.

## 3 RESULTS

### 3.1 Changes in myofibrillar protein content

**FIGURE 1:**
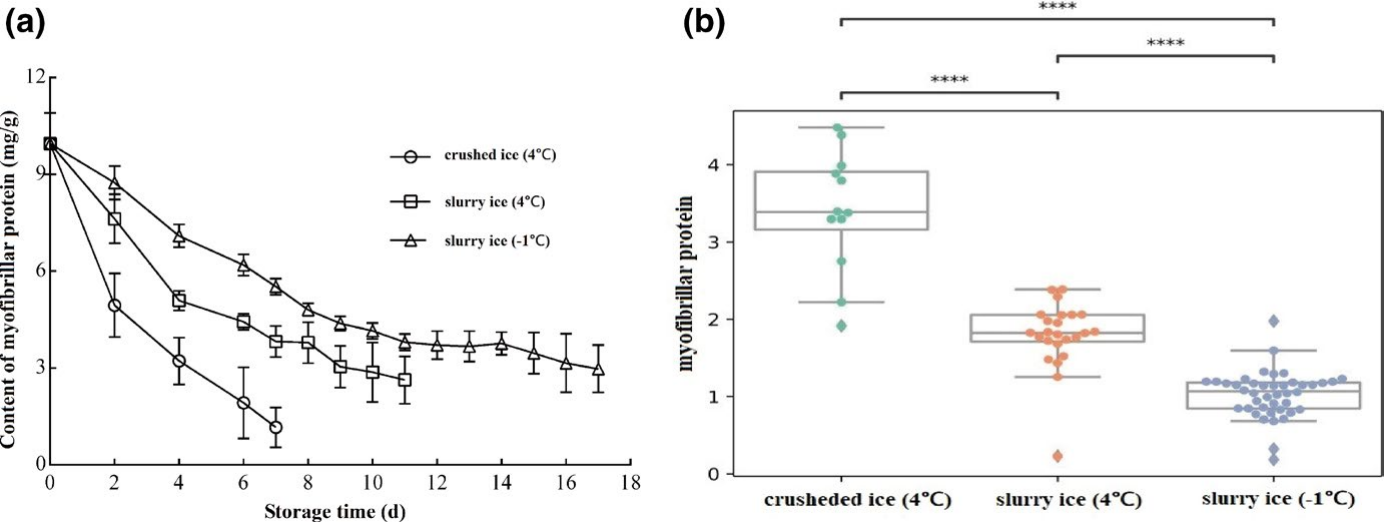
Effect of slurry ice on the content of myofibrillar protein of *Pseudosciaena crocea*

It was determined that the myofibrillar protein content of all three treatment groups of *P*. *crocea* showed a decreasing trend with the extension of storage time (Figure 1a). After 1 week of storage, the myofibrillar protein contents of *P*. *crocea* were 1.16 mg/g, 3.83 mg/g, and 5.53 mg/g in crushed ice (4℃), and slurry ice (4℃), and slurry ice (−1℃), respectively; compared with fresh samples, this equates to a decrease of 88.35%, 61.54%, and 44.48%, respectively. The protein content of fish stored in crushed ice (4℃) decreased the fastest, followed by slurry ice (4℃). During the late storage period, the amount of myofibrillar protein content in the slurry ice (4℃ and −1℃) group was 2.63 mg/g and 2.98 mg/g on days 11 and 17, respectively. This shows that slurry ice can slow down myofibrillar protein degradation and has a good refrigeration and preservation effect.

### 3.2 Changes in total sulfhydryl content

**FIGURE 2:**
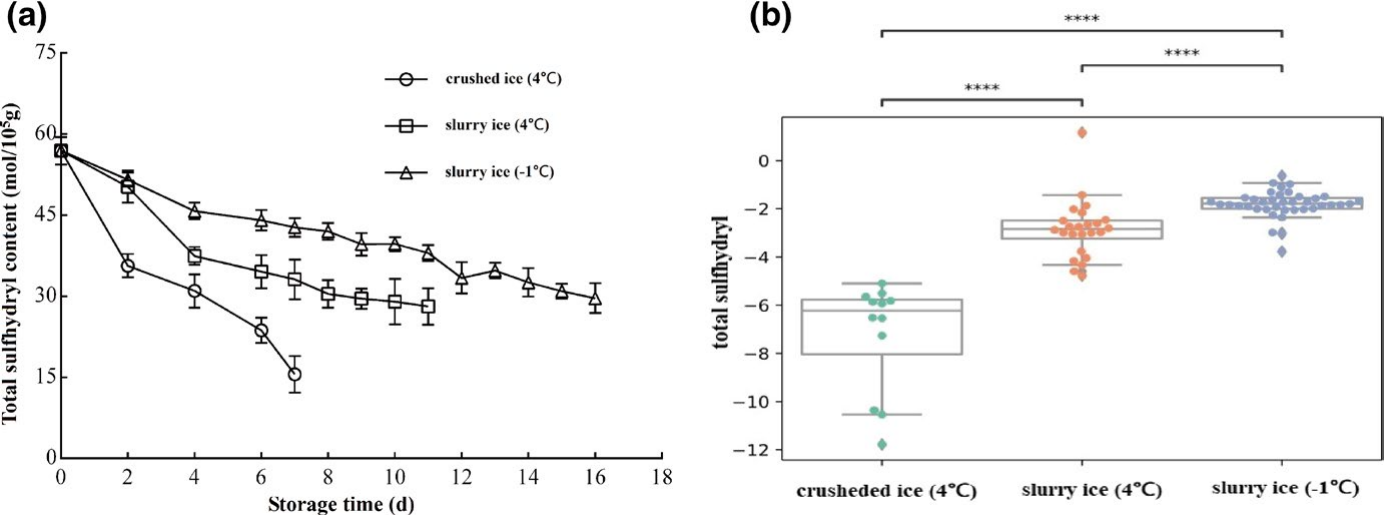
Effect of slurry ice on total sulfhydryl content in myofibrillar protein of *Pseudosciaena crocea*

Sulfhydryl groups are very active functional groups in the structure of fish protein, which can reflect the denaturation of protein to a certain extent. The total sulfhydryl content of fish meat in all three treatment groups decreased continuously with storage time (Figure 2a). The total sulfhydryl content of myofibrillar protein in fresh *P*. *crocea* was 56.96 mol/10^5^ g. The sulfhydryl content of fish stored on crushed ice (4℃) decreased the fastest to 15.62 mol/10^5^ g on the 7th day, which was 73.0% lower than the fresh fish. The total sulfhydryl content on the 7th day of slurry ice (4℃ and −1℃) was 33.16 mol/10^5^ g and 42.78 mol/10^5^ g, respectively. The results indicate that slurry ice treatment can slow down the decrease of total sulfhydryl content of myofibrillar proteins.

### 3.3 Changes in Ca^2+^‐ATPase activity

**FIGURE 3:**
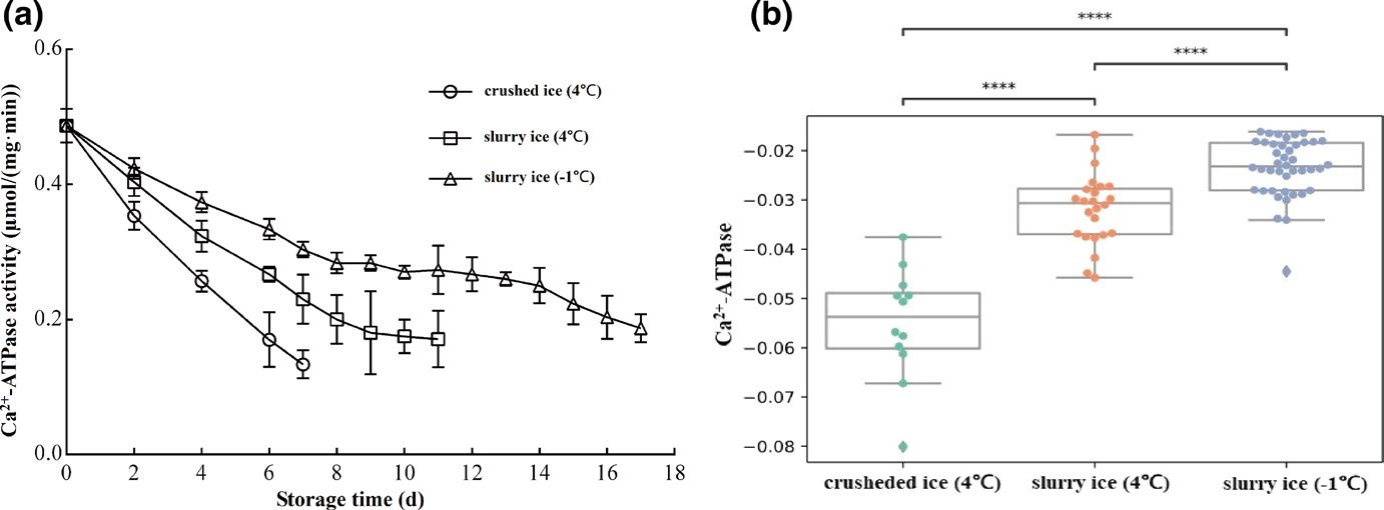
Effect of slurry ice on the content of myofibrillar protein Ca^2+^‐ATPase of *Pseudosciaena crocea*

The activity of ATPase is often used as an indicator to evaluate the protein denaturation of fish meat. The better the protein integrity, the better was the activity. The initial value (0 day) of Ca^2+^‐ATPase activity was 0.49 µmol/(mg·min) (Figure 3a). During the storage period, the Ca^2+^‐ATPase activity in all three treatment groups decreased while the Ca^2+^‐ATPase activity in the crushed ice group decreased at the fastest rate, reaching 73.5% on the 7th day. In the slurry ice (4℃) group, the Ca^2+^‐ATPase activity decreased rapidly from day 0 to day 8 and decreased by 59.2% on day 8. The rate of decrease slowed from day 8 and reached 0.17 µmol/mg·min on day 11. In the slurry ice (−1℃) group, there was only a 0.21 µmol/mg·min decrease from 0 to 11 days, and Ca^2+^‐ATPase activity was significantly higher than the control group. Thus, Ca^2+^‐ATPase activity in the *P*. *crocea* was better maintained using slurry ice treatment than broken ice.

### 3.4 Changes in surface hydrophobicity

**FIGURE 4:**
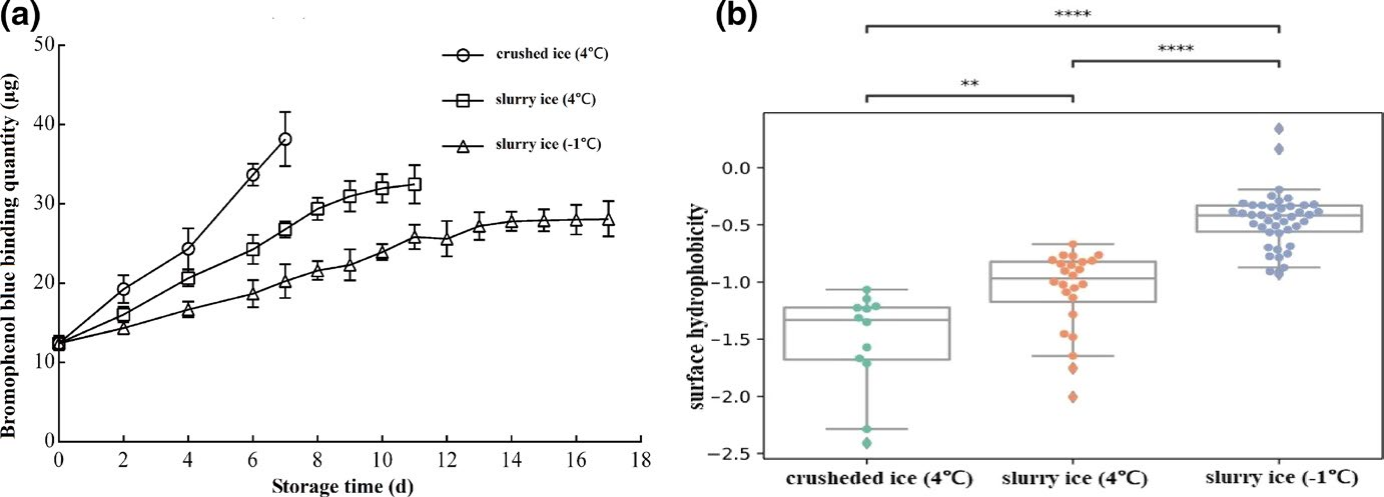
Effect of slurry ice on the surface hydrophobicity of myofibrillar protein

Surface hydrophobicity is one of the important structural properties of proteins. It can reflect the relative content of hydrophobic amino acids on the surface of protein molecules, which in turn indicates the degree of protein denaturation (Alizadeh‐Pasdar & Li‐Chan, 2000). The surface hydrophobicity of myofibrillar proteins of the fish increased in all three treatment groups with the extension of storage time (Figure 4a). The initial binding of bromophenol blue was 12.44 μg, and with the extension of storage time, the binding of bromophenol blue increased the fastest in the crushed ice group to 38.17 μg on the 7th day, which was 2.07 times higher than the initial binding. Storage on slurry ice (4℃ or −1℃) resulted in increases of 1.16‐ or 0.63‐fold on the 7th day, respectively. There was a significant linear correlation between surface hydrophobic values and storage time (R crushed ice (4℃) = 0.9881; R slurry ice (4℃) = 0.9878; R slurry ice (−1℃) = 0.9674).

### 3.5 Changes in TCA soluble peptides

**FIGURE 5:**
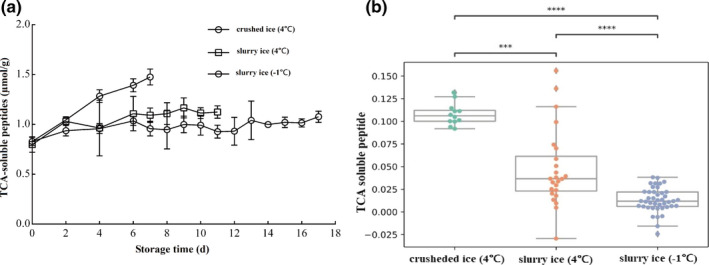
Effect of slurry ice on trichloroacetic acid (TCA)‐soluble peptides of *Pseudosciaena crocea*

The TCA soluble peptide content increased with the degree in protein degradation during storage. The TCA soluble peptide content of *P*. *crocea* in the three treatment groups increased with storage time (Figure 5a). There were 0.83 µmol/g TCA soluble peptides in the *P*. *crocea* sample on day 0, and there was a significant difference in TCA soluble peptide content between the control group and the slurry ice (4℃ and −1℃) group. From day 0 to 7, the amount of TCA soluble peptides increased rapidly with time in the control fish, and their contents were higher than those in slurry ice (4℃ and −1℃), indicating that the degradation rate of the fish was slower under slurry ice storage conditions, and the lower the storage temperature, the lower the degree of protein degradation.

### 3.6 Effect of slurry ice treatment on microstructure

**FIGURE 6:**
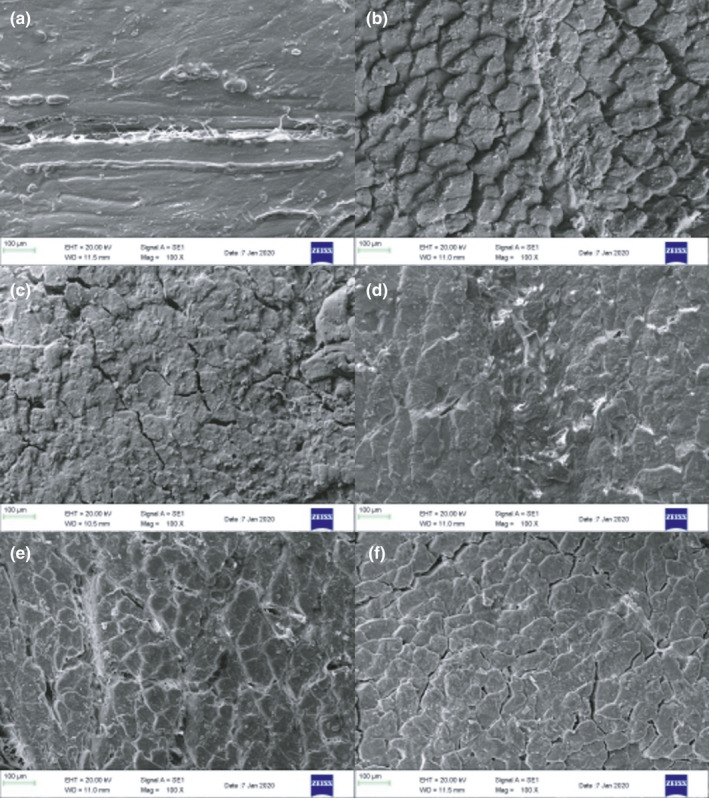
Scanning electron micrographs of muscle fibers under different storage conditions (a) untreated sample; (b) samples of crushed ice (4℃) group on the 6th day; (c) samples of slurry ice (4℃) group on the 6th day; (d) samples of slurry ice (−1℃) group on the 6th day; (e) samples of slurry ice (4℃) group on the 11th day; (f) samples of slurry ice (−1℃) group on the 11th day

The ultrastructure of fresh fish meat, and that stored under different refrigeration conditions was examined (Figure 6a‐f). The muscle fibers of fresh *P*. *crocea* are arranged in a compact manner, with no voids between the muscle fibers, and they have a flat and smooth surface (Figure 6a). On the 6th day, in the fish stored on crushed ice (4℃) and slurry ice (4℃), folds began to appear on the surface of the histones, and pores appeared (Figure 6b‐f). Muscle fiber separation in the fish meat in the crushed ice group was the most serious, while the muscle fiber arrangement was still more compact on the 6th day in the slurry ice (−1℃) group. On the 11th day, wrinkles and cracks on the surface of myofibrillar proteins in the slurry ice (4℃) group were more pronounced, and deep holes appeared, indicating that the protein degradation was severe, and that the structure was no longer intact. At the same storage time, the degree of muscle fiber separation was lower in the slurry ice (−1℃) group than in the slurry ice (4℃) group. This shows that slurry ice refrigeration can effectively slow down the degradation of collagen fibers, and that the lower the storage temperature, the slower the degradation rate of collagen fibers, and the better the cohesion in the fish tissue.

### 3.7 ANOVA

Storage treatments impacted the myofibrillar protein level (*p* <.05; Figure 1b). Pairwise comparisons among the three treatments indicate that each treatment was different from the others (*p* <.001). In particular, the −1℃ slurry ice had the lowest degradation rate for myofibrillar protein. Treatments also impacted the total sulfhydryl level (*p* <.05; Figure 2b). Pairwise comparisons among the three treatments indicate that each treatment was different from the others (*p* <.001). In particular, the −1℃ slurry ice has the lowest decay rate for total sulfhydryl content. Furthermore, Ca^2+^‐ATPase levels were impacted by the treatments (*p* <.05; Figure 3b). Pairwise comparisons among the three treatments indicate that each treatment is different from the others (*p* <.001). In particular, the −1℃ slurry ice has the lowest decay rate for Ca^2+^‐ATPase.

With regards to surface hydrophobicity, the three treatments had different effects (*p* <.05) (Figure 4b). Pairwise comparisons among the three treatments indicate that each treatment was different from the others. Comparison of the differences between the 4℃ crushed ice and 4℃ slurry ice treatments (0.01 < *p* <.05) was higher than that between other pairwise comparisons (*p* <.001). In particular, −1℃ slurry ice has the lowest decay rate for surface hydrophobicity. During the storage period, the surface hydrophobicity of fish protein in the slurry ice (4℃ and −1℃) treatment groups was lower than in the control group (*p* <.05), and the lower the storage temperature, the lower the hydrophobicity.

The different storage conditions impacted the TCA soluble peptide levels (*p* <.05; Figure 5b). Pairwise comparisons among the three treatments indicate that each treatment was different from the others. The difference between the 4℃ crushed ice and 4℃ slurry ice (*p* <.001) was greater than that between the other pairwise comparisons (*p* <.001), resulting from the several outliers ranging from 0.075 to 0.15 shown in the 4℃ slurry ice group. The −1℃ slurry ice was shown to have the lowest increase in TCA soluble peptides.

## 4 DISCUSSION

Protein oxidation is one of the major reasons for quality deterioration during storage of meat products (Zhang et al., 2013). The main oxidative modifications of proteins include sulfhydryl oxidation and the formation of carbonyl groups (Estévez, 2011; Liu et al., 2018). Sulfhydryl plays an important role in the structural characteristics of the myofibrillar protein (Han et al., 2019). Total sulfhydryl includes both those sulfhydryls uncovered on the exterior, i.e., reactive sulfhydryls, and those uncovered in the interior of the protein. In this study, the sulfhydryl content of the myofibrils of *P. crocea* meat in the three treatment groups decreased with an extension in storage time. The total sulfhydryl content of myofibrillar proteins decreases during storage, possibly because myofibrin is degraded and the spatial conformation of the protein is changed, so that the sulfhydryl groups buried in the interior of the molecule are exposed and oxidized, resulting in a decrease in sulfhydryl content (Benjakul et al., 2003). Sulfhydryl groups are the most reactive functional groups in myofibrillar proteins and are easily oxidized into disulfide bonds during storage (Chamba et al., 2014; Zhang & Ertbjerg, 2018). The results showed that the fish protein was gradually oxidized during the storage process, and this affected meat freshness and quality.

Glycolysis is a critical metabolic pathway in postmortem muscle providing a consistent ATP supply during the initial phase of the muscle to meat conversion (Liu et al., 2018). The activity of the Ca^2+^‐ATPase in myofibrils of *P*. *crocea* meat decreased in the three treatment groups with the extension in storage time. This suggests that glycolysis can play a key role in meat maturation processes, and with the extension of storage time, the muscles in the material produced a complex series of reactions, resulting in a decrease in muscle quality.

Hydrophobic fluorescence is increased in a nonpolar environment, and the change in surface hydrophobicity can reflect the change in myofibrillar protein in the microenvironment (Alizadeh‐Pasdar & Li‐Chan, 2000). The surface hydrophobicity increased and then slightly decreased with time (Figure 4). The increase in surface hydrophobicity can be attributed to the unfolding of the myofibrillar protein structure as more buried nonpolar amino acids are exposed to the surface of the myofibrillar protein (Lou et al., 2017). Denaturation of myofibrillar proteins leads to changes in the spatial conformation of proteins. The folding and gradual extension of myofibrillar protein molecules, and the exposure of hydrophobic groups inside the polypeptide chain, increases the hydrophobicity of the protein surface.

Tornberg (2005) showed that a good network structure is beneficial to the gel properties of protein. In this study, spaces between *P*. *crocea* muscle fibers gradually appeared with the extension in storage time, which may be because the collagen fibers of fish meat were gradually degraded under the action of enzymes and microorganisms, and the muscle fibers were gradually separated. This has also been reported to cause the phenomenon of "bone separation" in fish meat (Jiang & Wu, 2018). The degree of muscle fiber separation in the slurry ice treatment group was lower than that in the crushed ice group, indicating that the slurry ice refrigeration treatment could effectively alleviate the degradation of protein fibers; under slurry ice treatment, the lower the storage temperature, the slower the degradation rate of collagen fibers, and better the maintenance of the integrity of the fish tissue structure.

In this study, the myofibrillar protein content of *P*. *crocea* meat decreased with time under the three treatment conditions, and the myofibrillar protein in the fish meat was degraded due to the action of endogenous enzymes and spoilage microorganisms with the extension of storage time. The reactive sulfhydryls of myofibrillar proteins are oxidized to form disulfide bonds resulting in the polymerization of myosin heavy chains, which may also lead to a decrease in myofibrillar protein content (Zhang et al., 2012). The maintenance effect of *P*. *crocea* treated with slurry ice was better than that of the broken ice group (*p* <.05), indicating that the low temperature of the slurry ice system and the sealing protection could partially inhibit the enzyme activity and microbial growth and reproduction, slow down the oxidation process, reduce the deformation degree of myofibrillar protein, and thus maintain the freshness of the fish.

## 5 CONCLUSION

The effects of slurry ice were investigated in the meat of *P*. *crocea* (large yellow croaker fish). The results showed that with the extension of storage time, the myofibrillar protein, total sulfhydryl content, and Ca^2+^‐ATP enzyme activity decreased more than the control group, whereas the surface hydrophobicity was higher than that of the control group. At the same time, the electron microscopy observations found that slurry ice can maintain the integrity of muscle fiber tightness better. Therefore, the −1℃ slurry ice treatment was shown to be best cold storage condition in this study. Slurry ice can maintain the protein characteristics of large yellow croaker, retard quality reduction, and maintain the integrity of the fish tissue.

## CONFLICTS OF INTEREST

The authors declare that they have no known competing financial interests or personal relationships that could have appeared to influence the work reported in this paper.

## AUTHOR CONTRIBUTION

**Feng Guan:** Writing‐original draft (lead). **Yirui Chen:** Data curation (lead); Visualization (lead). **Simin Zhao:** Methodology (lead). **Zhuo Chen:** Writing‐original draft (supporting). **Chen Yu:** Methodology (supporting). **Yongjun Yuan:** Funding acquisition (lead); Supervision (lead).
